# Anomaly Detection Based on a 3D Convolutional Neural Network Combining Convolutional Block Attention Module Using Merged Frames

**DOI:** 10.3390/s23239616

**Published:** 2023-12-04

**Authors:** In-Chang Hwang, Hyun-Soo Kang

**Affiliations:** Department of Information and Communication Engineering, School of Electrical and Computer Engineering, Chungbuk National University, Cheongju-si 28644, Republic of Korea; inchang9507@gmail.com

**Keywords:** video surveillance, convolutional block attention module, area under the curve, equal error rate, computer vision, UBI-Fights, 3D convolution

## Abstract

With the recent rise in violent crime, the real-time situation analysis capabilities of the prevalent closed-circuit television have been employed for the deterrence and resolution of criminal activities. Anomaly detection can identify abnormal instances such as violence within the patterns of a specified dataset; however, it faces challenges in that the dataset for abnormal situations is smaller than that for normal situations. Herein, using datasets such as UBI-Fights, RWF-2000, and UCSD Ped1 and Ped2, anomaly detection was approached as a binary classification problem. Frames extracted from each video with annotation were reconstructed into a limited number of images of 3×3, 4×3, 4×4, 5×3 sizes using the method proposed in this paper, forming an input data structure similar to a light field and patch of vision transformer. The model was constructed by applying a convolutional block attention module that included channel and spatial attention modules to a residual neural network with depths of 10, 18, 34, and 50 in the form of a three-dimensional convolution. The proposed model performed better than existing models in detecting abnormal behavior such as violent acts in videos. For instance, with the undersampled UBI-Fights dataset, our network achieved an accuracy of 0.9933, a loss value of 0.0010, an area under the curve of 0.9973, and an equal error rate of 0.0027. These results may contribute significantly to solve real-world issues such as the detection of violent behavior in artificial intelligence systems using computer vision and real-time video monitoring.

## 1. Introduction

The mandatory installation of closed-circuit television (CCTV) in daycare centers and kindergartens owing to school violence and child abuse has been extensively debated, and the revision of these laws is being promoted as a precaution. Additionally, numerous intelligent CCTVs that detect or preempt dangerous situations early by analyzing the relationship between current and past situations in real time have been released. Modern intelligent CCTVs, capable of real-time situation analysis, play an integral role not only in deterring potential criminal activities but also in aiding in their resolution. Thus, the maintenance of safety and order is improved in the community via the CCTV recognition and classification of crime scenes. Particularly, the detection of assault situations is a key task that uses video anomaly detection. Anomaly detection, which has been the focus of several studies, is an algorithm that finds abnormal instances within the patterns of a specified dataset. The biggest challenge in the study of anomaly detection is that the dataset for these abnormal situations is smaller than that for normal situations. Even if the number of datasets increases, there remains a lack of data for completely abnormal situations. Recently, anomaly detection using unsupervised learning, that is, using unannotated datasets, has been increasing because data annotation is expensive and time-consuming. Many previous studies have used unsupervised learning, such as generative adversarial network (GAN) [1] and autoencoder [2], using two-dimensional (2D) convolution. However, anomaly detection using multi-instance learning is also a popular trend. In this study, a model was constructed using a three-dimensional (3D) convolutional neural network (CNN), which is the most commonly used supervised traditional deep learning method. 3D CNNs have 3D filters and significantly more parameters; however, because fewer pre-trained models exist compared to 2D CNN, training often starts from the ground up without weights. Therefore, 3D CNNs appear unsuitable for constructing and learning sufficiently deep structures. However, space-time data can be considered simultaneously, which enables effective recognition and classification of dynamic behavior, including violent behavior. Because our model uses a 3D CNN, it can learn both spatial and temporal data, and the convolutional block attention module (CBAM) [3] is used to identify the most crucial information, thereby improving the interpretability and focus of the model. The model can extract features from various dimensions, thereby contributing to the recognition and classification of complex behavioral patterns.

While other 3D CNNs use multiple frames as input simultaneously, we merged multiple frames into one image and used multiple images as input. Therefore, even if the same number of images was input, memory usage was reduced, which was useful for environments with large datasets or memory limitations. Consequently, substantial amounts of temporal data could be used simultaneously, enabling more precise detection of anomalous behavior. Important features could be extracted and learned more effectively by aggregating multiple information frames from one location. This improved the recognition of complex behavioral patterns and could increase the accuracy of violent behavior detection. Additionally, using input data in this manner is similar to the method of light field imaging [4], which acquires 3D information by combining images captured from various angles. Light field imaging is yet to prove effective at detecting abnormalities. However, in this study, we generated data in a way similar to light-field imaging and extracted temporal patterns by merging continuous frames to generate 3D information at various points. Our method was inspired by light-field imaging, and it aims to improve precision in anomaly detection.

In contrast to traditional methods that utilize 2D images or video data, this data-merging method uses a complex input structure, in which multiple frames are contained in one image. Therefore, the form of the input data in a CNN can significantly affect the performance of the model, indicating the value of investigating various input methods in this manner.

Finally, this form of input data did not previously exist, and it may be helpful in the study of anomaly detection. Because it is similar to inputting images by dividing them into patch units, such as with a vision transformer (ViT) [5], future research could expand upon this idea. Therefore, in this study, we seek to emphasize the effect of the input data structure on the CNN performance and pave the way for anomaly detection techniques that expand on this structure. The findings of this study indicate that compared to existing state-of-the-art methods, our approach exhibits superior performance, which enables improved focus on areas of interest. Furthermore, rather than assessing a situation by observing a static scenario, such as a 2D CNN, our method can be applied to various scenarios, such as robbery, arson, and theft using the correlation between the past, present, and future. Additionally, in this paper, experiments are conducted in an environment that has relatively few obstacles and does not obscure actions, rather than a highly enclosed environment.

## 2. Related Works

Anomaly detection is the most popular and challenging topic in the field of computer vision, and it entails several methods, including 3D convolution, weakly supervised learning, unsupervised learning, skeleton, and optical flow. This section introduces these and other methods.

### 2.1. Three-Dimensional Convolution

Inspired by deep learning innovations in the rapidly developing image domain, 3D convolution (C3D) [6] can extract temporal and spatial information, in contrast to 2D convolution. The model is trained using a combination of dropout, batch normalization, and data augmentation techniques. The deep structures in the model perform better than the shallow structures, highlighting the importance of capturing long-term structures in videos. C3D has effective features for detecting anomalies, and several studies have been conducted on it [7,8,9,10].

#### 2.1.1. Exploring Kernel Temporal Depth

In [6], C3D with a kernel size of d×k×k was tested by changing only the kernel depth, where d is the kernel temporal depth and k is the kernel space size. In this network, eight convolution layers, five pooling layers (a pooling layer appears immediately after each convolution layer), two fully connected layers, and a softmax loss layer were used for predicting action labels. All convolution layers had the same kernel temporal depth and four networks were set with kernel temporal depths of 1, 3, 5, and 7 for testing. The kernel temporal depth with the best performance was 3×3×3.

#### 2.1.2. Spatiotemporal Feature Learning

Deconvolution is used to understand what C3D learns internally. C3D starts by focusing on the shapes in the first few frames and then tracks the prominent behavior in the following frames. Detecting shapes and movements simultaneously is the most noticeable difference between C3D and 2D CNN.

### 2.2. Weakly Supervised Learning

Currently, few datasets are available for anomaly detection, and the resulting annotation task requires a significant investment of time and money. Therefore, although frame-level labels have been used in supervised learning in the past, many studies have recently been conducted on anomaly detection via weakly supervised learning using labels at the video level [11,12,13,14,15]. Weakly supervised learning saves time and money, and the related multi-instance learning is used extensively.

#### 2.2.1. Multi-Instance Learning

Multi-instance learning (MIL) is a variant of supervised learning in which a single class label is assigned to a set of instances such as image patches. Therefore, while each instance is not labeled for prediction, multiple instances form a bag, which is labeled for prediction. This is one of the most widely used techniques because it requires less annotation, is suitable for weakly supervised learning, and can learn the labels of each instance (snippet or frame) using annotations at the entire video level.

MIL [16,17,18,19,20] is useful for identifying the specific parts of various snippets in a video where an abnormality occurs. Additionally, it significantly reduces the misclassification of snippets or frames that are normal. Because it focuses on certain parts of the video, abnormalities can be detected in greater detail. In [18], we considered the video as a background, where each frame was divided into multiple instances, generating instance scores via MIL, prior to performing anomaly detection. Similarly, ref. [20] used a framework that treated a video as a bag and video clips as instances of a bag, using both normal and abnormal video clips. In [16], a self-learning framework was also used.

#### 2.2.2. Gaussian Mixture Model

In [15], the Gaussian mixture model (GMM) modeled data distributions using probability distributions. Data distribution is identified by the combination of several Gaussian distributions. Thus, GMM expresses data distribution as the sum of the weights of several Gaussian distributions.

In video anomaly detection, the GMM models change over time at the pixel level, and changes in brightness or color values for each pixel are modeled as Gaussian distributions to distinguish between normal and abnormal patterns. An anomalous pattern is one that deviates significantly from the general Gaussian distribution. Degardin [15] proposed Bayesian filtering and GMM-based approaches for anomaly detection in video images under an MIL paradigm, similar to weakly supervised learning. This method predicts and measures the high anomaly score for a video segment in an anomalous situation. Experiments were conducted on the UBI-Fights, UCF-Crime, and UCSD Ped1 datasets, with an area under the curve (AUC) of 0.906 and equal error rate (EER) of 0.160, an AUC of 0.759 and EER of 0.302, and an AUC of 0.801 and EER of 0.236, respectively.

### 2.3. Unsupervised Learning

Unsupervised learning is a learning method that does not use any labels, in contrast to weakly supervised learning that uses a limited number of labels, owing to the constraints of available anomalous datasets on anomaly detection. Unsupervised learning methods include the k-means clustering algorithm [21], which uses Euclidean distances; one-class support vector machine (SVM) [22,23]; GAN [24,25,26,27]; and autoencoder [28,29,30]. Several unsupervised learning methods are described below.

#### 2.3.1. K-Means Clustering and One-Class SVM

K-means clustering [31] is an unsupervised machine learning algorithm that divides a dataset into K nonoverlapping and distinct clusters. Doshi et al. [21] applied a fast unsupervised anomaly detection system using K-means clustering consisting of three modules responsible for pre-processing, candidate selection, and backtracking anomaly detection. This method significantly reduced the overhead during test operations and eliminated the overhead during learning operations.

A one-class SVM [23] is a model that focuses on the decision boundary of normal data when insufficient amounts of anomalous data exist, and it learns to detect anomalies based on this decision boundary. In contrast to SVM, because the hyperplane is divided based on the origin, there can be only one class regardless of the quantity of data and the severity of the normal/anomalous imbalance. The model achieved an AUC of 94.3 and EER of 10.0 at the frame level on the UCSD Ped1 dataset and an AUC of 70.3 and EER of 34.0 at the pixel level. Additionally, for the entrances of the Subway Entrances and Exits dataset, it achieved an AUC of 90.5 and EER of 16.9, whereas, for the exits, it recorded an AUC of 90.8 and ERR of 15.4.

#### 2.3.2. Generative Adversarial Networks

GANs [1] were first introduced by Goodfellow et al. in 2014. Well-trained GANs, known for their ability to produce samples that are nearly indistinguishable from real data, have also been extensively studied for anomaly detection applications.

In 2017, Ravanbakhsh and Nabi [24] proposed a method for detecting strange events in videos using a GAN. Using this method, the generator learns the distribution of normal videos, and the discriminator detects anomalies based on this information. This study is significant for the application of GAN to video anomaly detection, and it has significantly influenced subsequent studies.

In 2018, Wang et al. [23] proposed an anomaly detection method based on future video frame prediction. The key concept of this study is a model that predicts future video frames from normal sequences and detects anomalies by comparing real and anticipated frames. This study set a new standard in the field and influenced subsequent works [32,33]. Additionally, Li et al. [33] proposed, for a given video frame, first creating continuous frames before and after using a two-branch generator network and then minimizing the prediction error between the generated frames and their real-world counterparts. They evaluated three datasets: CUHK Avenue, UCSD Ped2, and Shanghai Tech Campus. Furthermore, several other studies have been conducted, including [25,34].

#### 2.3.3. Autoencoder

Autoencoders were popularized in 2006 by Hinton and Salakhutdinov [2]. A well-learned autoencoder can generate the same input data as that of a GAN.

Wang et al. [28] proposed a shallow generation network consisting of two neural networks to obtain models such as a Gaussian mixture that fit the distribution of real data. This model has contributed to maximizing the advantages of CNN and variational autoencoder. Experiments were conducted on UCSD, Avenue, UMN, and PETS datasets, and a high performance was achieved on all except UCSD.

Chong and Tay [29] proposed an autoencoder structure comprising 3D convolution layers. Therein, if generation was not properly performed while reproducing an image in the autoencoder, abnormalities were detected. Experiments were conducted on Avenue, UCSD Ped1 and Ped2, and Subway Entrances and Exits datasets. AUC and EER were used as evaluation indicators, and top performance was achieved on the subway exits, UCSD Ped1, and Avenue data.

### 2.4. Other Methods

In this section, we describe additional network methods such as skeleton-based, optical flow-based, ViT, and convolution long short-term memory (ConvLSTM) as well as those based on 3D CNN such as inflated 3D CNN (I3D).

#### 2.4.1. Skeleton-Based Method

In the fields of computer vision and deep learning, the human body is mainly represented by the skeleton, that is, bones and their connections (joints). Skeletal data are used to track, analyze, recognize, and model human behavior, and human movements are represented by specific patterns or sequences. Anomalous behavior can be considered a deviation from these patterns.

Morais et al. [35] presented a new method for detecting anomalies using skeletal features in normal human behavior patterns. In [35], the authors proposed a network called Message-Passing Encoder–Decoder Recurrent Neural Network (MPED-RNN) and evaluated it using the ShanghaiTech Campus and CUHK Avenue datasets. In the ShanghaiTech dataset, 101 videos related to humans are classified as human-related (HR) ShanghaiTech. The authors achieved the highest performance on the HR ShanghaiTech and ShanghaiTech datasets with receiver operating characteristic area under the curve (ROC AUC) scores at the frame level of 0.754 and 0.734, respectively. Similarly, the Avenue dataset was divided into HR Avenue and Avenue, and ROC AUC scores were obtained at frame levels of 0.863 and 0.862, respectively.

Du et al. [36] stated that skeleton-based motion recognition is generally considered a time series problem and pointed out the deficiencies of existing methods, such as temporal pyramids (TP) and hidden Markov models (HMMs). To overcome these shortcomings, the author proposed a hierarchical recurrent neural network (RNN) that not only combined human physical structures and behavior patterns but also extracted temporal dynamic representations of skeletal sequences by maximizing deep RNN in modeling long-term context information of time sequences. Experiments on the Berkeley MHAD and Motion Capture Database HDM05 datasets showed that the best performance was achieved on both with 100% and 96.92% accuracy, respectively.

#### 2.4.2. Optical Flow

An optical flow is a vector field that represents the movement of a pixel in an image over time by indicating the extent and direction of its movement. Assuming that the brightness of a pixel is constant over time and that adjacent pixels have similar movements, the direction and size of the pixel can be expressed as a vector by calculating its movement between two consecutive frames. Abnormalities are detected in subtle movement differences in rapid, repetitive, or complex situations, rather than in patterns of general movement.

In 2019, Nguyen and Meunier [37] proposed a model with an autoencoder structure that converts video frames into optical flows. Liu et al. [32] uses an optical flow that is optimized based on the adversarial loss between the optical flow and original output video frame, and the optical flow can be extracted directly from a single frame because FlowNet [38] is built inside the network. The model learns the spatial structure for normal data via a Conv-AE and then determines the association between the pattern of each input data in the second stream and the corresponding motion represented by the optical flow. Furthermore, optical flows extracted from FlowNet2 [39] detected discontinuous behavior well owing to clear boundaries and comparison of the optical flow prediction with the actual answer values; it achieved the highest performance on the CUHK Avenue and UCSD Ped2 datasets. However, as in [24], it has the drawback of time collisions.

Wang et al. [40] proposed a new approach to overcome these shortcomings. Owing to the significant discrepancy between the ground truth and predicted optical flow, which causes blurring in abnormal areas, optical flow with duality for both normal and abnormal data was used to predict the next frame from the current frame. Therefore, for normal behavior, the optical flow makes the predicted frame close to the real frame; however, for anomalous behavior, noise is added. They experimented with UCSD Ped2, Avenue, and ShanghaiTech datasets, achieving AUC scores of 94.2, 88.4, and 75.3, respectively, with Avenue and ShanghaiTech exhibiting the highest performance.

#### 2.4.3. Vision Transformer

The transformer was first proposed by [41] to provide an alternative to RNN- and CNN-based natural language processing models. Additionally, in [42] they proposed a bidirectional encoder presentation from transformers (BERT), which achieved state-of-the-art performance in several natural language processing (NLP) tasks by pre-training transducers for unlabeled text in both directions.

A ViT divides the image into several equally sized patches, each of which is planarized into vectors that have position embeddings. The output of the transformer is passed to the head for classification, and during training, prediction uses the output of the last layer. Additionally, ViT can process continuous frames as time-series data and is useful for learning the relationships and patterns between frames.

Yuan et al. [43] proposed an approach for video anomaly detection using a ViT, which introduces a self-attention mechanism to overcome the inherent locality of convolutional operations and the difficulty of modeling long-range relationships when reconstructing a GAN or an autoencoder. The author used a video ViT (ViViT)- [44] and U-Net- [45] based approach called TransAnomaly, which solves vision problems based on the transformer. After decoding the features using U-Net, abnormalities were detected using the difference between the predicted and actual frames [46]. Their modified ViViT enabled the effective encoding of input images in all dimensions of space-time. The performance of the model was evaluated using the AUC. For UCSD Ped1 and Ped2 and Avenue datasets, they obtained AUCs of 0.867, 0.964, and 0.870, respectively, representing the best performance on the UCSD Ped1 and Avenue datasets.

#### 2.4.4. ConvLSTM

ConvLSTM is a variant of long short-term memory (LSTM), an RNN effective at processing sequential data, which incorporates convolutional operations. ConvLSTM is particularly useful for processing time-series data while preserving spatial structure information by performing convolutional operations instead of matrix operations within the LSTM. It was first introduced in 2015 by Shi et al. [47] to solve weather forecasting problems. ConvLSTM is suitable for anomaly detection tasks because it can process spatial (information within frames) and time series features (information between frames) simultaneously to extract spatio-temporal features and learn dynamic patterns in video sequences.

Luo et al. [48] proposed a method for integrating ConvLSTM with an autoencoder called ConvLSTM-AE to create a common CNN capable of learning the regularity of typical shapes and movements. Similar to the other methods, anomaly detection was performed using frames regenerated in the decoder; however, abnormalities can be detected only in continuous frames. An experiment was conducted using the Moving-Mnist dataset, which was divided into application and motion parts. The results showed that the models with ConvLSTM-AE or ConvLSTM performed better than convolutional autoencoder (Conv-AE), achieving AUCs of 0.999 and 0.949, respectively, in the appearance and motion sections. Experiments were also conducted on Avenue, UCSD Ped1 and Ped2, and Subway Entrances and Exits, with AUCs of 0.70, 0.755, 0.881, 0.933, and 0.877, respectively, achieving the best performance on all datasets.

Medel and Savakis [49] proposed an approach for video anomaly detection using several stacked ConvLSTMs capable of end-to-end learning. The overall model structure was an autoencoder structure similar to the previous models, and three ConvLSTM layers were used inside the encoder. The evaluation was conducted using the USCD Ped1 and Ped2, Subway Entrances and Exits, and Avenue datasets. True positive/false positive and precision/recall were used as evaluation indicators. For each dataset, the true positive/false positive results were 40/7, 12/1, 62/14, 19/37 (anomalies: 19), 29/15 (anomalies: 30), and 40/2, and the precision/recall results were 0.851/1.00, 0.923/1.00, 0.816/0.939, 0.339/1.00, 0.659/0.967, and 0.952/0.957, respectively.

#### 2.4.5. I3D

I3D was first proposed in 2017 by Carreira and Zisserman [50]. I3D is a 3D extension of the existing 2D CNN inflation structure where 3D represents height, width, and time, that is, the 3D convolution of the 2D CNN inflation. I3D processes information by considering the temporal continuity between video frames such that it allows existing 2D convolutional filters and pooling layers to process (inflate) video data in 3D. For example, if the size of the 2D convolution filter is k×k, it is expanded to 3D and converted into k×k×k. I3D can learn the actions and patterns between consecutive frames by adding temporal dimensions. Thus, the motions and patterns of the videos are recognized more accurately. By extending the weight of the 2D filter to 3D, the initial value effectively learns the 3D network. I3D also proposes a principle that uses two streams: RGB and optical flow. The RGB stream processes the frames of the original video by focusing on the visual and color information, and the optical flow stream determines the movements and patterns of objects in a video. By combining these two streams, I3D can recognize behavior in a video more accurately. Improving the accuracy of motion recognition using both visual and motion information is a major feature of I3D. Additionally, anomaly detection studies continue to be published [51,52,53,54,55,56,57,58,59,60].

## 3. Proposed Method

In this section, we propose a network architecture that correctly classifies violent situations by using the spatial and temporal features of each video to detect abnormal situations, particularly violent ones, and achieves state-of-the-art performance. The proposed architecture adds a convolutional block attention module (CBAM) [3], an attention module that includes the channel attention and spatial attention modules, to a residual neural network (ResNet) [61] in the form of a 3D convolution.

### 3.1. CBAM (Convolutional Block Attention Module)

CBAM is a network module used to highlight the channel and spatial information within a convolutional block. This improves the performance of CNNs and is commonly used in image classification and various computer vision tasks, highlighting important information. It comprises two attention modules.

**Figure 1:**
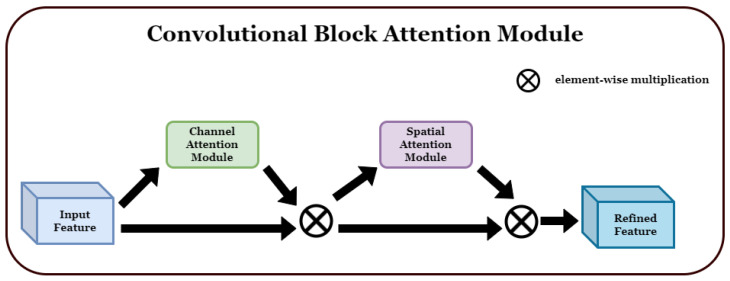
Overview of the convolutional block attention module (CBAM): It consists of a channel attention module and a spatial attention module [3].

**Figure 2:**
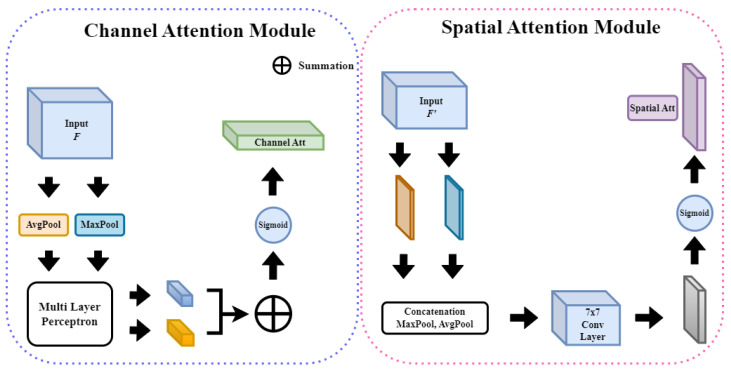
Structure of each attention sub-module of CBAM [3]: Divided into channel attention and spatial attention. Output is generated after passing max pooling and average pooling.

1. Channel attention: Channel attention determines the importance of each channel and plays a role in emphasizing the relationships between channels. Thus, important features can be emphasized, and unnecessary noise can be suppressed. Channel attention is usually implemented by using the average value of each channel, calculated via global average pooling, to learn the relationships between channels. 2. Spatial attention: Spatial attention plays an important role in understanding the spatial structure of feature maps by emphasizing one area and suppressing another. To this end, the importance of the feature map calculated for each channel was determined, and a spatial weight was generated and multiplied by the feature map. If the input feature map *F* is multiplied by $M_{C}$, which is the channel attribute map, $F^{\prime }$ is obtained. If it is multiplied by $M_{S}$, which is the spatial attribute map, $F^{\prime \prime }$ is obtained, which is the final feature map. Figure 1 shows the structure of the detailed sub-module of CBAM, and the formulas for obtaining $M_{C}$ and $M_{S}$, which are the channel attention map and spatial attention map, respectively, are shown as below Equations (1) and (2) in below. Figure 2 shows the structure of each attention sub-module of CBAM.

(1) $$\begin{array}{c} M_{C}\left(F\right)=\sigma \left(MLP\left(AvgPool\left(F\right)\right)+MLP\left(MaxPool\left(F\right)\right)\right)\\ =\sigma (W_{1}\left(W_{0}\left(F_{avg}^{C}\right)\right)+W_{1}\left(W_{0}\left(F_{max}^{C}\right)\right)) \end{array}$$

(2) $$\begin{array}{c} M_{S}\left(F\right)=\sigma \left(f^{7\times 7}\left(\left[AvgPool\left(F\right);MaxPool\left(F\right)\right]\right)\right)\\ =\sigma (f^{7\times 7}\left(\left[F_{avg}^{S};F_{max}^{S}\right]\right)) \end{array}$$

The channel attention map calculates the importance of each channel of an input feature map and extracts the relationships between the channels. After calculating the attention score and passing through average and max pooling, two vectors are produced, which are input into the shared multilayer perceptron to add nonlinearity. This is then encoded with a probabilistic value between zero and one using a sigmoid function to generate an $M_{C}$, one-dimensional 1×1×C channel attention map. If the input feature map *F* is multiplied by $M_{C}$, $F^{\prime }$, which is a feature map to which the channel attachment is applied, is generated. A spatial attention map is created by encoding the pixels to be focused on per C×H pixels throughout the channel. Up until the pooling process and concatenation, it is the same as channel attention. This information is gathered for comprehensiveness because both pooling methods are carried out to extract spatial information and calculate the importance of location. Additionally, this value is placed in the 7×7 conv2d layer and a 2D spatial attention map is generated via a sigmoid function. The feature map generated in this manner is of size H×W×1. When the resulting $M_{S}$ is multiplied by $F^{\prime }$, the final attention map is obtained, that is, $F^{\prime \prime }$ is produced. The formulae for $F^{\prime }$ and $F^{\prime \prime }$ as follows Equations (3) and (4):

(3) $$F^{\prime }=M_{C}\left(F\right)⊗F$$

(4) $$F^{\prime \prime }=M_{S}\left(F^{\prime }\right)⊗F^{\prime }$$

We introduce the proposed method by transforming the attention-based approach proposed in [62,63]. In this paper, we describe how the output of a linear classification layer can be influenced in addition to a popular CNN pipeline that has excellent feature extraction capabilities. CBAM influences the feature extraction process of the model and serves to weigh the network output for improved feature extraction.

### 3.2. Grid Frame

In this section, we describe how to create a grid frame for each dataset and how to assign labels accordingly.

In this study, we propose a method to improve the input data processing methods of existing 2D and 3D CNNs. In general, 2D CNNs adopt a method of processing one image at a time. Consequently, each image is processed independently by considering only the spatial features and thus does not consider the correlation between images. Therefore, to detect abnormalities using 2D CNNs, an RNN such as LSTM or a neural network structure such as ConvLSTM should be added. However, the proposed method of processing multiple images of 3D CNNs at once includes temporal and spatial features. Thus, this method has the advantage of considering correlations between images.

Additionally in this section, we present an approach that extends beyond these image-processing methods, where for each dataset, several images are processed in a single group. This group consists of continuous frames, and each image is processed considering its interrelationship with other images in the group, rather than being processed independently. This enables the model to extract more information and meaningful patterns than a single image of a 2D CNN or multiple images of a 3D CNN, thereby enabling accurate prediction and analysis. This approach aims to capture and use complex relationships between multiple images in a group (this can include, for example, multiple groups corresponding to a video) and finally detect abnormalities by identifying spatial and temporal patterns via the continuity of images in and between groups. This approach addresses complex interactions between images and groups, enabling the derivation of more accurate and meaningful results. Furthermore, this study opens up the possibility of new methods in the field of anomaly detection using an input method conceptually similar to Lightfield and ViT. In this study, we propose inputs of sizes 3×3, 4×3, 4×4, and 5×3.

For the UBI-Fights dataset, each annotation file marked fighting or nonfighting frames with a 1 or 0, respectively. Therefore, when preprocessing videos identified as fights, the annotation file was loaded and frame indices with a value of 1 were saved. The frame indices were read at intervals of 5. For example, if frames 1–20 had a value of 1, then frames 1, 6, 11, and 16 were accessed. As the video has a frame rate of 30 fps, without this interval, significant action changes may not be captured; hence, spacing between frames is necessary. Once sufficient frames were gathered for the intended composite image size (9 frames for 3×3, 15 frames for 5×3, and so on), they were combined into a single composite image. Owing to memory constraints, only ten composite images were generated per video. These ten merged images served as the input data, and depending on the size of the composite image, 90–150 frames were used from a single video. Data corresponding to the normal class were processed in the same manner to generate the input data. This method was adopted and used on all datasets, including UBI-Fights.

The UBI-Fights dataset showed an imbalance between the fight (216) and normal (784) class data. Therefore, experiments were conducted not only at the existing data ratio fight:normal = 216:784 but also by equalizing the data to fight:normal = 216:216, resulting in two experiments. Furthermore, experiments were conducted separately using ResNet depths of 10, 18, 34, and 50, with the data split in an 80:15:5 ratio for training:validation:test datasets.

The RWF-2000 dataset was balanced, and because the training to validation split was already at an 8:2 ratio, only one experiment was conducted. This dataset does not have separate annotations; therefore, the Violence and NonViolence categories were arbitrarily assigned values of 1 and 0, respectively, for experimentation. In contrast to the UBI-Fights dataset, given that the videos were 5 s long with 30 fps, after reading the video, the frames were extracted in the flow of the video to create merged images. This was performed under the assumption that sufficient actions corresponding to the classes were contained within.

**Figure 3:**
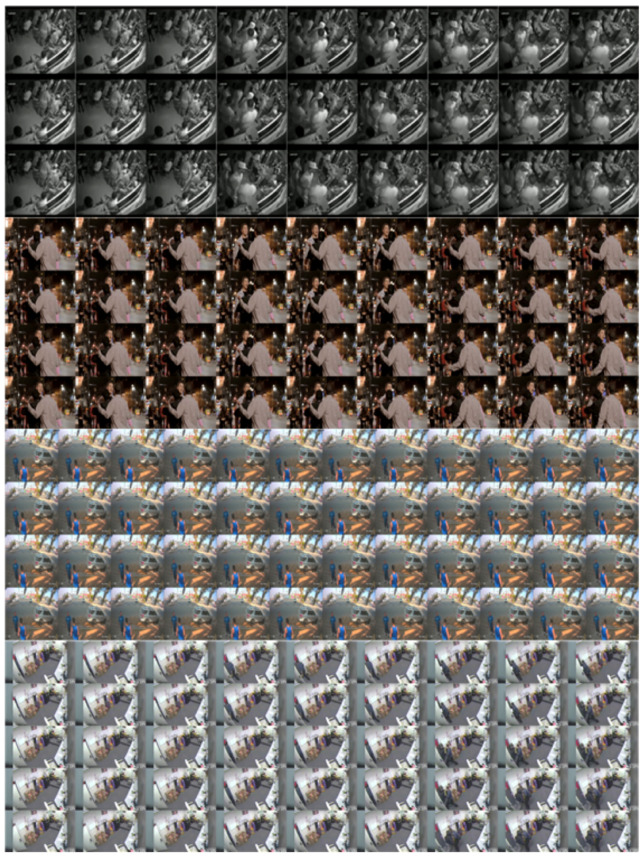
Example of each grid frame: Frames go from top left to top right, and from bottom left to bottom right. They have the order of 3×3, 4×3, 4×4, and 5×3 inputs in order from the top, and all are samples of three consecutive images. The total number of frames used in the example is 27, 36, 48, and 45 in order.

UCSD Ped1 originally contained normal data in the training set and anomalous data in the test set; however, they were arbitrarily mixed for use. Similarly, Ped2 was mixed arbitrarily. For both datasets, the ratio of the training and test sets was the same as that in the original datasets, and the test set was used for validation. Furthermore, only a randomly selected 30% of the test set was used for testing. For the UCSD Ped1 and Ped2 datasets, we used all the data. A sample of each input data is described in Figure 3.

### 3.3. Model Architecture

Recently, various studies have been conducted on abnormal detection using CBAM, such as [62,63,64]. However, most research has been conducted on structures such as 2D CNN-based generative models. This study employs a different approach to propose a more effective anomaly detection model. Our work differs from other studies because it effectively combines 3D CNN and CBAM for anomaly detection. Additionally, instead of using an autoencoder structure, our approach optimizes the characteristics of 3D CNN and the effects of CBAM and proposes a more sophisticated and robust anomaly detection model. Thus, our study presents an original perspective as compared with previous studies.

#### 3.3.1. Backbone Architecture

In this study, ResNet [61] was used as the backbone architecture. This is a suitable choice for its efficacy in learning deep neural networks. Particularly, the problem of gradient loss can be solved despite the depth of the model using the advantages of residual blocks. Residual blocks assist in the effective transfer of gradients via residual connections between inputs and outputs and minimize the loss of information, even in deep networks. Furthermore, deeper and more complex models can be developed. Subsequently, the detailed structure of the model and connections between the layers are described.

#### 3.3.2. Proposed Architecture

The model architecture proposed in this study was constructed by adding CBAM after each bottleneck (or basic block) of 3D ResNet. This configuration may improve anomaly detection performance by effectively combining the feature extraction capabilities of the existing 3D CNN with the information emphasis capabilities of CBAM. CBAM can be placed behind each bottleneck, and the model can be extended to include additional residual blocks as required. This increases the expressiveness of the model and aids in capturing complex temporal relationships.

CBAM is an attention mechanism designed primarily for 2D data. However, as this study dealt with 3D data, the code was appropriately modified. First, 2D pooling (AvgPool2d and MaxPool2d) was replaced with AdaptiveAvgPool3d and AdaptiveMaxPool3d, which perform pooling by automatically adjusting kernel and stride sizes to fit the target output tensor sizes. They also work with inputs of various sizes and produce the same output. As channel attention aims to calculate the mean and maximum values for the entire spatial dimension, using adaptive pooling to obtain the outputs of (1, 1, 1) is appropriate. Adaptive pooling also performs pooling to the desired output size without a fixed kernel size or stride, which can help optimize the computing resources. Finally, the overall information in the input feature map was averaged to minimize information loss while maintaining a consistent output size.

(1) Channel Attention (designed to recognize and learn the importance of each channel)
  (a) Adaptive average and max pooling: This module aggregates the spatial information of 3D data. Average and max pooling each return a tensor with a size of C×1×1, where *C* is the number of channels in the input tensor.
  (b) MLP with Conv3d: Importantly, the kernel sizes of both the first and second Conv3d layers are 1×1×1. The first Conv3d layer serves to reduce the number of channels from *C* to C/reductionratio. Conversely, the second Conv3d layer increases the number of channels from the C/reductionratio to *C* again.
(2) Spatial attention (designed to learn the importance of each 3D location)
  (a) Max and min pooling: Extracts the maximum and minimum values of the channel at each location of the input data. This results in a tensor of size 2×D×H×W. Here, *D*, *H*, and *W* are depth, height, and area, respectively.
  (b) Three-dimnsional convolution: The tensor generated above passes through the 3D convolution layer, which has a kernel size of 7×7×7 and a number of output filters (channels) of 1. A larger kernel size accounts for more amount of spatial information in convolutional operations, with a 7×7×7 size kernel accounting for a large area of its surroundings at each 3D location, which aids in capturing a broader context. The output channel is 1 to generate a spatial attention map. This attention map has the same spatial dimension as the original input tensor but only represents the importance (or attention score) of each position. Therefore, the number of output filters is 1, which is multiplied by the original input tensor and element-wise to apply spatial attention. In summary, the setting with a kernel size of 7×7×7 and a number of output channels of 1 aims to recognize a wide range of contexts, save parameters, and create a clear spatial attention map.

When the input data are provided, 3D convolution is performed, and the CBAM layer is passed through batch normalization, rectified linear unit (ReLU), and dropout. After passing through all the reserved blocks, the feature map undergoes global average pooling through avgpool. This leaves only the average value for each feature map, and the feature after pooling is a 1D vector, which passes through the fully connected layer (fc layer) and outputs the final predicted value to perform anomaly detection.

In detail, the input data go through 7×7×7 convolution, batch normalization, ReLU, and MaxPooling, and they are then input to the bottleneck layer (in the case of ResNet50) or basic block (in case of ResNet 10, 18, and 34). Here, after going through 1×1×1 convolution, batch normalization, ReLU, 3×3×3 convolution, batch normalization, ReLU, 1×1×1 convolution, and batch normalization, and lastly, reduction in computational complexity when the output and input sizes of the bottleneck are different owing to the difference in stride or number of input and output channels. Downsampling (stride = 2) is then performed for high-level feature extraction. Subsequently, CBAM is applied and channel attention and spatial attention proceed. Layers containing bottlenecks or basic blocks are repeated four times from layers 1 to 4, and CBAM is applied to each bottleneck block or basic block. In the case of ResNet 10, 18, and 34, there are (1, 1, 1, 1), (2, 2, 2, 2), and (3, 4, 6, 3), respectively, basic block layers in layer 1. In the case of ResNet50, there are 3, 4, 6, and 3 bottleneck layers in layer 1 to layer 4, respectively. After the input data have executed all the network structures, classification for anomaly detection is performed in the fully connected layer.

**Figure 4:**
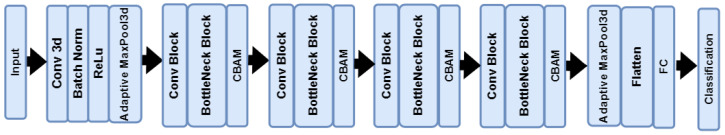
Proposed Architecture.

In the architecture of the proposed network, when an input arrives, it passes through Conv3d, batch normalization, ReLU, and AdaptiveMaxPool3d and then through the convolutional block (there are 1×1, 3×3, and 7×7 depending on the depth of the model) and the basic block (or bottleneck block). Subsequently, the process of going through CBAM is repeated four times before proceeding with the classification. In Figure 4, it is labeled as “BottleNeck” based on ResNet50, but for shallower depths (10, 18, 34), it changes to “BasicBlock”.

### 3.4. Dataset

In this section, we describe datasets that we used for experiments. When looking at existing datasets for anomalous behavior detection, few datasets contain violent actions. While there are numerous datasets like UMN [65], UCSD Ped1 and Ped2 [66,67], Subway Entrances and Exits [68], and CUHK Avenue [69], the anomalous behaviors they designate refer to actions like walking in a different direction from the majority in a crowd or riding bicycles and inline skates. There are also datasets such as [70] that occur in public transportation, especially buses. However, these datasets contain many obstacles in public transportation and there are many cases where these obstacles obscure human actions. Because we only tested frames where human actions were clearly visible, we also excluded this dataset from the experiments. Hence, a dataset that clearly distinguishes between violent and nonviolent behaviors is required. Therefore, this study used UBI-Fights [71] and three other datasets.

#### 3.4.1. UBI-Fights

The UBI-Fights dataset was divided into fight and normal classes, consisting of 216 and 784 videos, respectively, gathered from YouTube and LiveLeak based on extensive text search results of violent incidents. The data were collected at the frame level with annotations totaling 80 h in length; however, the length of each video varied and had a frame rate of 30.00 frames per second with a fixed resolution of 640×360. A drawback of the videos in the fight class is that the entire duration of the video does not show fighting; hence, scenes that should be categorized as normal were included.

#### 3.4.2. RWF-2000

Another dataset used was the RWF-2000 [72]. This dataset consists of 2000 video clips filmed in the real world by surveillance cameras, each 5 s long at 30.00 frames per second. They were also sourced from YouTube and divided into violent and nonviolent classes. Unlike in [71], the training and test sets were divided in an 8:2 ratio. The resolution of the videos varied, including 240p, 320p, 720p, and 1080p. An advantage of this dataset was that the actions were aligned with their respective classes throughout the video. Therefore, this assumption was more intuitive. The violence class included fights, robberies, explosions, shootings, bleeding, and assaults, enabling a more detailed categorization depending on the situation and purpose. However, because the resolutions varied, preprocessing through resizing could alter the original size of videos, potentially leading to a loss of information.

#### 3.4.3. UCSD Ped1 and Ped2

We included the UCSD Ped1 and Ped2 datasets in our experiment, which considered behaviors, such as walking in the opposite direction to the majority in a crowd, riding a bicycle, or using inline skates, as anomalous. The ground truths of UCSD Ped1 and Ped2 are provided in the form of a segmentation map in which objects exhibiting anomalous behaviors are highlighted in white. The UCSD Ped1 dataset consists of images from 70 videos: 34 for training and 36 for testing. Each set contains 200 images of size 158×238. The training set comprises normal behaviors, whereas the testing set contains 10 anomalous behaviors. The UCSD Ped2 dataset comprises images from 28 videos, with 16 for training and 12 for testing. Similar to UCSD Ped1, each image contains 200 images of size 360×240. Consequently, any area in the segmentation map with a value of 1, is interpreted as representing abnormal behavior and assigned a label of 1; otherwise, it is assigned a label of 0.

### 3.5. Details of Training

In this section, we describe the optimizer and loss function used and the hardware and software environments and so on. As this study is a binary classification, the loss function used was BCEWithLogitsLoss. The equation for BCEWithLogitsLoss is as follows in Equation (5):

(5) $$l\left(x,y\right)=\left\{\begin{array}{cc} -w\cdot (1-y)\cdot \log(1-\sigma (x)) & \mathrm{if}\;y=0\\ -w\cdot y\cdot \log(\sigma (x)) & \mathrm{if}\;y=1 \end{array}\right.$$

where σ represents the sigmoid function, ω stands for the weight for each input sample, and *y* indicates the actual label value (0 or 1). The loss of y=1 (positive class) measures the accuracy of the model prediction using the cross-entropy term, whereas the loss of y=0 (negative class) measures the inaccuracy of the model prediction. In both cases, the closer the prediction is to the actual label, the closer the loss becomes to 0. This function takes the logits (that is, the outputs before passing through the sigmoid activation function) and actual label values as inputs. This effectively combines a two-step operation (sigmoid activation function and binary cross-entropy loss). This merging method was used to achieve computational efficiency and numerical stability.

In this study, the ResNet architectures with 10, 18, 34, and 50 layers were utilized as the foundational backbones. Following the addition of the Convolutional Block Attention Module (CBAM) on four occasions within each architecture, the total number of layers increased to 14, 22, 38, and 54, respectively. For optimization, AdamW was used. AdamW, a variant of the Adam optimization algorithm, implements weight decay separately. This is particularly beneficial in preventing overfitting and enhancing the generalization performance in deep neural networks. The combination of 3D ResNet and CBAM, which forms a profound network architecture, benefits from AdamW for efficient learning and regularization. An initial learning rate of 5 $\times 10^{-3}$ was adopted. In complex tasks such as anomaly detection, setting the appropriate learning rate is crucial. A starting learning rate of 5 $\times 10^{-3}$ is high enough to facilitate rapid convergence, yet it is not so high as to risk overfitting or training instability. And the learning rate was adjusted using the ReduceLROnPlateau scheduler. This scheduler reduces the learning rate automatically when there is no improvement in validation loss. Learning rate can be optimized through training and adjusted. This prevents the model from becoming trapped in local minima during training and aids in fine-tuning performance through more delicate adjustments. Adjusting the learning rate during training is especially crucial in complex tasks like anomaly detection. Callback functions (Early Stopping) were also employed to prevent overfitting by halting the training if the validation loss did not decrease for a certain period. While the epoch count was initially configured to 100, the actual number is different due to the activation of callback functions. Owing to memory constraints, the batch size was set to one. Experiments were conducted on a Windows 10 operating system with hardware specifications of an 11th Gen Intel(R) Core(TM) i7-11700K with a 3.60 GHz CPU, 48.0 GB RAM, and an NVIDIA GeForce RTX 4090 GPU. All the proposed architectures were implemented using PyTorch.

### 3.6. Evaluation Metrics

In this section, we describe evaluation metrics for experiments. The evaluation metrics used were accuracy (ACC), loss, AUC, and EER. ACC is one of the most intuitive metrics used in classification problems and represents the ratio of items correctly classified by the model to the total number of items. Accuracy is follow as Equation (6):

(6) $$Accuracy=\frac{Number\;of\;Correct\;Predictions}{Total\;Number\;of\;Predictions}=\frac{TP+TN}{TP+TN+FP+FN}$$

In the context of model evaluation, the loss function quantifies the discrepancy between the predicted values of the model and the actual data. A lower loss value signifies that the model’s predictions are more closely aligned with the true values. Furthermore, the Receiver Operating Characteristic (ROC) curve, plotted with the False Positive Rate (FPR) on the x-axis and the True Positive Rate (TPR) on the y-axis, serves as a critical tool for assessing classification performance. The area under the ROC curve (AUC) is particularly instrumental, providing a numerical benchmark for performance evaluation. The AUC value, ranging from 0 to 1, reflects the model’s effectiveness; values approaching 1 indicate superior model performance as the curve nears the upper left corner of the graph. The formulations for TPR and FPR are delineated in Equations (7) and (8), respectively.

(7) $$TPR=\frac{TP}{TP+FN}$$

(8) $$FPR=\frac{FP}{FP+TN}$$

EER is the error rate at the point where the false acceptance rate (FAR) and false rejection rate (FRR) intersect. In other words, in the graph, ERR denotes the error rate at the point where the FAR and FRR cross (become equal). A value closer to 0 indicates a better performance of the classification model. FRR defined as follows Equation (9):

(9) $$FRR=\frac{FN}{TP+FN}$$

## 4. Results

In this section, we list all the experimental results of the proposed method for each dataset. Additionally, we present the results of various existing experiments and perform analyses and comparisons of these results. All figures are rounded off to the fifth decimal place. Two experiments were conducted for UBI-Fights. Before conducting the experiment, we addressed the class imbalance issue in the dataset by undersampling the fight class. Subsequently, the first experiment was conducted using the adjusted dataset, and the second experiment was performed using the original. For RWF-2000 and UCSD Ped1 and Ped2, only one experiment was conducted without altering the data ratio.

### 4.1. UBI-Fights

In this section, we introduce the experimental results for the UBI-Fights dataset. The UBI-Fights dataset consists of videos in two categories: fight and normal, with 216 and 784 videos, respectively. The size of the merged images used in the experiment was 800×800.

Table 1 presents the experimental results for the UBI-Fights dataset with undersampling applied. For 3×3, 4×3, 4×4, and 5×3 inputs, the networks based on ResNet 18, ResNet 34, ResNet 10, and ResNet 10, respectively, demonstrated the highest performance. This suggests that if a model, such as ResNet 50, is too deep and complex, it may face classification difficulties. We observed challenges in feature extraction when the model became excessively deep or when a certain number of frames were used on images of limited size.

Table 2 lists the experimental results for the original UBI-Fights dataset. For 3×3, 4×3, 4×4, and 5×3 inputs, the networks based on ResNet10, ResNet18, ResNet10, and ResNet10, respectively, demonstrated the highest performance. Because undersampling was not performed, the amount of data increased, leading to superior performance in relatively lightweight networks. Additionally, for the 4×4 input, both ResNet10 and ResNet18 excelled in the two evaluation metrics. However, because the primary evaluation metrics for the UBI-Fights dataset are AUC and EER, the ResNet10-based network can be considered slightly more dominant.

As shown in Table 3, compared with other methods, our approach employed two different experimental methods based on data distribution. We chose the method with higher AUC and EER values because the remaining models were only compared based on AUC and EER. Notably, the method with 3×3 input achieved an AUC of 0.9973 and an EER of 0.0027. Similarly, most of our methods surpassed state-of-the-art results. Particularly, by observing the results from the 3×3 and 4×3 based on ResNet10 and ResNet18 backbone models, it is clear that fewer images were used in the merged image, and relatively lower model complexity(depth) yielded better outcomes.

### 4.2. RWF-2000

This section presents the experimental results for the RWF-2000 dataset. Because the original RWF-2000 data ratio was split as 8:2 for the training and testing, only one experiment was conducted. Additionally, there were 2000 videos, with 800 for training and 200 for testing each for the Violence and Nonviolence categories. As shown in Table 4, similar to UBI-Fights, as the complexity of the backbone network increased, the performance tended to deteriorate. Compared with UBI-Fights, it is evident that the performance is generally higher. Table 5 presents a comparison of recent studies using the traditional RWF-2000 dataset.

Table 4 lists the experimental results for RWF-2000. For 3×3, 4×3, 4×4, and 5×3 inputs, the networks based on ResNet10, ResNet18, ResNet10, and ResNet10 demonstrated the highest performance. Because the primary evaluation metric for this dataset was accuracy, in the case of the 4×3 input, ResNet18 was considered superior.

Table 5 compares the proposed method with other approaches using the RWF-2000 dataset. Because accuracy is the primary evaluation metric for this dataset, we prioritized it when comparing performances. Compared with other methods, the network using a 3×3 input configuration achieved the highest performance with an accuracy of 0.9920.

### 4.3. UCSD Ped1

In this section, we present the experimental results for the UCSD Ped1 dataset. Only the abnormal data have ground truth in the form of segmentation maps, in which the anomalies are segmented in white. If there is even one pixel with a value of 1 in the ground truth image, a label of 1 is assigned; otherwise, a label of 0 is assigned. For our experiment, we randomly added a portion of the anomalous data from the test dataset to the training set and proceeded with the training.

Table 6 lists the experimental results for the UCSD Ped1 dataset. For 3×3, 4×3, 4×4, and 5×3 inputs, the networks based on ResNet18, ResNet34, ResNet18, and ResNet10 demonstrated the highest performance. Overall, using ResNet18 as the backbone network with a 3×3 input achieved the highest performance, with an AUC of 0.9188 and an EER of 0.1197. However, this record is ranked second highest following an adversarial generator [24] (Table 7).

For the UCSD Ped1 dataset, the method with a 3×3 input achieved an AUC of 0.9188 and an EER value of 0.1197. However, this was the second-highest record, following the Adversarial generator [24].

### 4.4. UCSD Ped2

In this section, we present experimental results for the UCSD Ped2 dataset. The training dataset contained only normal data, whereas the test dataset contained only abnormal data. Thus, we randomly added a portion of the abnormal data from the test set to the training set for the experiment. The ground truth was provided in the same manner as UCSD Ped1; therefore, the labeling method and subsequent training were conducted in the same way.

Table 8 presents the experimental results for UCSD Ped2. For 3×3, 4×3, 4×4, and 5×3 inputs, the networks based on ResNet10 demonstrated the highest performance. The best performance was achieved with a 3×3 input using ResNet10 as the backbone network, resulting in an AUC of 0.9959 and an ERR of 0.0081.

Table 9 compares the proposed approach with other methods. Because other methods primarily assessed performance based solely on AUC, we assigned the highest priority to AUC for comparison. The network using the 3×3 format achieved the second-best performance, with an AUC of 0.9959, slightly below that of the state-of-the-art model.

## 5. Conclusions and Future Research

In this study, we introduce a 3D CNN-based network combined with an attention module for real-time anomaly detection, particularly for violent behavior detection and video surveillance, and a unique input data approach. This network has several unique features compared with those of previous studies and uses state-of-the-art deep learning technology and attention mechanisms to effectively extract the features of temporal and spatial information in the context of the data. A comparison of our method with state-of-the-art methods demonstrates the superior performance of our approach, which enables improved focus on areas of interest. Rather than assessing a situation by observing a static scenario, such as a 2D CNN, our method can be applied to various scenarios, such as robbery, arson, and theft, if desired, by using the correlation between the past, present, and future. Additionally, in this study, an input video with multiple consecutive frames in one image, called a grid frame, was applied to the network with cbam to simultaneously view spatiotemporal features from multiple viewpoints. This approach enabled a shift from simple 2D image analysis to dynamic video analysis.

However, there remains the challenge of memory constraints. Because of memory constraints, the number of frames applied to a single video was limited. To address this issue, we are currently exploring methods such as reducing the size of images using grid frames or diminishing the complexity of 3D convolutional models. To circumvent the memory limitations restricting the number of frames per video, it is essential to reduce either the image size during data preprocessing or the duration of each video in the dataset. For instance, segmenting a 10 s video into ten 1 s clips ensures the availability of complete data information, irrespective of memory constraints. So, addressing this issue in the data preprocessing step significantly will improve the classification performance owing to the increased input data. We believe that this network will lead to significant breakthroughs in anomaly detection. This study is expected to advance research on the application of the attention module to 2D CNN. If these models can be efficiently lightweight, it holds promise for real-time detection applications in industrial settings. Furthermore, it is anticipated that the application of this model will not be limited solely to crimes occurring between individuals. This study can serve as a basis for research on potentially hazardous areas, such as manufacturing plants, food inspection sites, and construction sites, where dangerous situations can occur instantaneously.

To improve results, it is expected that the results of all the used datasets can be improved by performing transfer learning on the network used in this paper or through data augmentation and ensemble techniques. A second approach is to use multimodal data, adding other types of data, such as audio. This is expected to improve results for this dataset by allowing the detection of anomalies that are difficult to identify using visual data alone.

Finally, future studies will apply this network to broader datasets to ensure the best classification performance, regardless of the input data method used. We will also continue to work to better detect anomalous behavior in datasets with deeper occlusion and public transportation and to achieve good results on larger and more complex datasets. We expect that this study will be used in various fields and will inspire further research directions.

## Figures and Tables

**Table 1:** Experimental results on datasets with undersampling applied.

Input	Backbone	ACC	LOSS	AUC	EER
3×3	ResNet10	0.9925	0.0022	0.9970	0.0030
	**ResNet18**	**0.9933**	**0.0010**	**0.9973**	**0.0027**
	ResNet34	0.9858	0.0068	0.9943	0.0060
	ResNet50	0.8025	0.2368	0.8625	0.1991
4×3	ResNet10	0.9419	0.0174	0.9747	0.0293
	ResNet18	0.9680	**0.0009**	0.9881	0.1510
	**ResNet34**	**0.9762**	0.0069	**0.9905**	**0.0099**
	ResNet50	0.8625	0.1789	0.7952	0.2037
4×4	**ResNet10**	0.9534	**0.1804**	**0.7932**	**0.2588**
	ResNet18	**0.9595**	0.2032	0.7516	0.2707
	ResNet34	0.9535	0.3222	0.7254	0.3045
	ResNet50	0.9535	0.3778	0.6392	0.3780
5×3	**ResNet10**	0.9789	0.0352	**0.9363**	**0.0615**
	**ResNet18**	**0.9980**	**0.0286**	0.8021	0.1789
	ResNet34	0.8551	0.2153	0.8006	0.1989
	ResNet50	0.7851	0.3265	0.7978	0.2060

**Table 2:** Experimental results on the original dataset.

Input	Backbone	ACC	LOSS	AUC	EER
3×3	**ResNet10**	**0.9916**	**0.0002**	**0.9817**	**0.0224**
	ResNet18	0.9913	0.0071	0.9345	0.0737
	ResNet34	0.9720	0.0211	0.8668	0.1418
	ResNet50	0.8344	0.2397	0.8259	0.1834
4×3	ResNet10	0.9419	0.0174	0.9747	0.0293
	**ResNet18**	**0.9680**	**0.0009**	**0.9881**	**0.0151**
	ResNet34	0.9270	0.0004	0.9647	0.0487
	ResNet50	0.8358	0.2867	0.7952	0.0950
4×4	**ResNet10**	**0.9799**	0.1732	**0.8411**	**0.2187**
	ResNet18	0.9799	**0.1715**	0.8406	0.2324
	ResNet34	0.9798	0.4752	0.8194	0.2481
	ResNet50	0.9799	0.4776	0.6637	0.3357
5×3	**ResNet10**	**0.9767**	0.1017	**0.9363**	**0.0969**
	ResNet18	0.9489	**0.0433**	0.7772	0.2231
	ResNet34	0.9500	0.0460	0.7075	0.3036
	ResNet50	0.9500	0.1480	0.6066	0.3952

**Table 3:** Comparison of recent studies. Table 3 compares the results of recent studies using the UBI-Fights dataset. There is only one model with an AUC exceeding 90, and in most cases, the EER is also greater than 0.2.

Method	AUC	EER
SS-Model + WS-Model + Sultani et al. [73]	0.846	0.216
SS-Model [73]	0.819	0.284
GMM [15]	0.906	0.160
Sultani et al. [19]	0.787	0.267
s2 VAE [28]	0.610	0.427
LSTM-AE [29]	0.541	0.480
Adversarial generator [24]	0.533	0.484
**Grid frame (ours) (3×3)**	**0.9973**	**0.0027**
Grid frame (ours) (4×3)	0.9905	0.0099
Grid frame (ours) (4×4)	0.8411	0.2187
Grid frame (ours) (5×3)	0.9363	0.0615

**Table 4:** Experimental results on the RWF-2000 dataset.

Input	Backbone	ACC	LOSS	AUC	EER
3×3	**ResNet10**	**0.9920**	**0.0211**	**0.9962**	**0.0059**
	ResNet18	0.9884	0.0343	0.9363	0.1418
	ResNet34	0.8596	0.0386	0.8548	0.1436
	ResNet50	0.8846	0.2449	0.6006	0.3586
4×3	ResNet10	0.9784	0.0177	**0.9962**	**0.0487**
	**ResNet18**	**0.9787**	**0.0026**	0.9330	0.0677
	ResNet34	0.8764	0.3460	0.8800	0.0976
	ResNet50	0.8579	0.3524	0.8732	0.1158
4×4	**ResNet10**	**0.9506**	**0.0029**	**0.9071**	**0.1572**
	ResNet18	0.9015	0.0343	0.9001	0.1756
	ResNet34	0.8687	0.4059	0.8059	0.1989
	ResNet50	0.7350	0.4734	0.7000	0.2700
5×3	**ResNet10**	**0.9506**	**0.0029**	**0.9550**	**0.0583**
	ResNet18	0.9382	0.0071	0.9071	0.0972
	ResNet34	0.8532	0.3023	0.7734	0.1789
	ResNet50	0.8004	0.3087	0.7900	0.2139

**Table 5:** Comparison with other methods using the RWF-2000 dataset.

Method	ACC
Structured keypoint pooling [74]	0.934
Semi-supervised hard attention (SSHA) [75]	0.904
Human skeletons + change detection [76]	0.9025
Separable convolutional LSTM [77]	0.8975
SPIL convolution [78]	0.893
Flow-gated network [72]	0.8725
**Grid frame (ours) (3×3)**	**0.9920**
Grid frame (ours) (4×3)	0.9787
Grid frame (ours) (4×4)	0.9506
Grid frame (ours) (5×3)	0.9506

**Table 6:** Experimental results using the UCSD Ped1 dataset.

Input	Backbone	AUC	EER
3×3	ResNet10	0.8828	0.1880
	**ResNet18**	**0.9188**	**0.1197**
	ResNet34	0.8988	0.1606
	ResNet50	0.8787	0.1849
4×3	ResNet10	0.8846	0.1875
	ResNet18	0.8846	0.1890
	**ResNet34**	**0.9047**	**0.1597**
	ResNet50	0.8717	0.1915
4×4	ResNet10	0.8721	0.1913
	**ResNet18**	**0.8846**	**0.1836**
	ResNet34	0.8503	0.1985
	ResNet50	0.8726	0.1875
5×3	**ResNet10**	**0.8846**	**0.1875**
	ResNet18	0.8830	0.1884
	ResNet34	0.8824	0.1881
	ResNet50	0.8652	0.1936

**Table 7:** Comparison with other methods on the UCSD Ped1 dataset.

Method	AUC	EER
S2-VAE [28]	0.876	-
sRNN [79]	0.8171	-
ConvAE [29]	0.899	0.125
GMM [15]	0.801	0.236
**Adversarial generator [24]**	**0.974**	**0.008**
Grid frame (ours) (3×3)	0.9188	0.1197
Grid frame (ours) (4×3)	0.9047	0.1597
Grid frame (ours) (4×4)	0.8846	0.1875
Grid frame (ours) (5×3)	0.8846	0.1875

**Table 8:** Experimental results using the UCSD Ped2 dataset.

Input	Backbone	AUC	EER
3×3	**ResNet10**	**0.9959**	**0.0081**
	ResNet18	0.9953	0.0111
	ResNet34	0.9000	0.1666
	ResNet50	0.7875	0.2134
4×3	**ResNet10**	**0.8750**	**0.2000**
	ResNet18	0.8457	0.2040
	ResNet34	0.8730	0.2006
	ResNet50	0.7969	0.2121
4×4	**ResNet10**	**0.8672**	**0.2025**
	ResNet18	0.8594	0.2051
	ResNet34	0.8613	0.2006
	ResNet50	0.8262	0.1895
5×3	**ResNet10**	**0.8750**	**0.2057**
	ResNet18	0.8574	0.2000
	ResNet34	0.8691	0.2000
	ResNet50	0.8449	0.2011

**Table 9:** Comparison with other methods using the UCSD Ped2 dataset.

Method	AUC	EER
Background-agnostic framework [80]	0.987	-
RTFM [14]	0.986	-
CL-VAD [81]	0.978	-
SSMTL [82]	0.975	-
FastAno [83]	0.963	-
GCN-Anomaly [84]	0.932	-
AI-VAD [85]	0.991	-
Adversarial generator [24]	0.935	0.14
**DMAD [86]**	**0.997**	-
Grid frame (ours) (3×3)	0.9959	0.0081
Grid frame (ours) (4×3)	0.8750	0.2000
Grid frame (ours) (4×4)	0.8672	0.2025
Grid frame (ours) (5×3)	0.8750	0.2057

## Data Availability

The datasets used in this paper are public datasets.
